# Modified argon laser therapy for benign tumor of the eyelid

**DOI:** 10.1186/s12886-022-02601-w

**Published:** 2022-09-24

**Authors:** Jisang Han, Shin-Hyo Lee, Chul Young Choi, Ramin Khoramnia, Jaemin Kim, Hyun Jin Shin

**Affiliations:** 1 Department of Ophthalmology, Kangbuk Samsung Hospital, Sungkyunkwan University School of Medicine, Seoul, Republic of Korea; 2 Department of Oral Anatomy and Histology, College of Dentistry, Kyung Hee University, Seoul, Republic of Korea; 3 Department of Ophthalmology, University of Heidelberg, Heidelberg, Germany; 4 Department of Medicine, Graduate School, Kyung Hee University, Seoul, Korea; 5 Department of Ophthalmology, Research Institute of Medical Science, Konkuk University Medical Center, Konkuk University School of Medicine, Seoul, Republic of Korea

**Keywords:** Argon laser, Benign tumor, Eyelid, Treatment, Stain

## Abstract

**Background:**

To report about the therapy of benign eyelid tumors with a modified argon laser technique as an alternative to surgery.

**Methods:**

Nineteen benign tumors of the eyelid were included in this study. After staining the surface of the tumor with a violet marker, low-energy argon laser photoablation was performed. A mean number of 312 spots (spot size ranging from 150 to 500 μm) with a power of 200 to 400 mW, and a duration between 0.1 and 0.2 s were applied.

**Results:**

The eyelid tumors were located mainly in the lower eyelid (58%). Dermal nevi and papilloma were the most frequently treated lesions. Over a mean follow-up period of 10.5 months (range 6–18 months), all eyelid tumors were successfully treated by a single session of laser therapy. All patients were satisfied with the laser therapy and the cosmetic result. No postoperative complications were observed. No relapses occurred during follow-up.

**Conclusions:**

Our modified method of argon laser therapy utilizes the staining of the surface of the eyelid tumor to increase the amount of thermal laser energy absorbed by the target. This novel technique is simple and effective for treating benign eyelid tumors.

**Supplementary Information:**

The online version contains supplementary material available at 10.1186/s12886-022-02601-w.

## Introduction

Up to 85% of eyelid tumors are benign lesions [1]. The most common treatment for benign tumors of the eyelids is surgical excision. However, eyelid surgery is not always uneventful. Complications such as eyelid malposition, eyelid notching, and contracture scars can sometimes occur [2, 3]. Laser treatment is a less invasive technique than surgery, which is particularly of interest in cases with cosmetic indications such as benign eyelid lesions [4, 5]. Also, it could be an alternative in patients with multiple systemic diseases or poor physical conditions, because it avoids the inconvenience of a traditional surgical approach.

The argon laser is one of the most commonly used lasers in ophthalmology. Therefore, it is available in most ophthalmology clinics. The spectral range of the argon laser has emission peaks at wavelengths of 488 and 514 nm. Photocoagulation of tissue occurs when light energy is transformed into thermal energy [5]. The argon lasers are used in a variety of ophthalmic surgeries (e.g., removal of conjunctival cysts, iridotomy, and trabeculoplasty) due to its convenience, effectiveness, and safety [6–9]. Also, Wohlrab et al. [10] reported cases in which benign tumors of the eyelid were successfully removed with an argon laser.

We previously reported a modified method of removing conjunctival cysts using an argon laser, staining the surface with markers, which increases the absorption of thermal laser energy on the targeted lesion with good results [11]. In this regard, staining the surface of an eyelid mass with a dark-purple marker could also be helpful when using an argon laser for the treatment of benign eyelid tumors. Thus, we describe a novel method and results for removing eyelid tumors using an argon laser rather than a conventional surgery.

## Materials and methods

Medical records of 19 patients with benign eyelid tumors treated with argon laser were reviewed at Konkuk University Hospital between December 2014 and January 2020. The study followed the tenets of the Declaration of Helsinki and was approved by the Institutional Review Board/Ethics Committee at Konkuk University Medical Center. Informed consent was obtained from each patient before the procedure.

All patients included in the study wished to undergo the procedure for cosmetic reasons. Before the treatment and during follow-up, the patient underwent a comprehensive ophthalmic examination, including visual acuity, intraocular pressure, and slit lamp examination. Only patients with lesions that did not show any signs of malignancy were treated according to the method used in this study.

The benign tumors of the eyelid were divided into small (≤ 5 mm), and large (> 5 mm) lesions based on the average of vertical and horizontal diameters. At the last visit, patients were asked if they were satisfied with the procedure. Treatment was considered successful when the mass had been completely removed without recurrence and the patient was satisfied with the cosmetic results during the follow-up visit. Only patients with a follow-up period of 6 months or more were included.

**Fig. 1:**
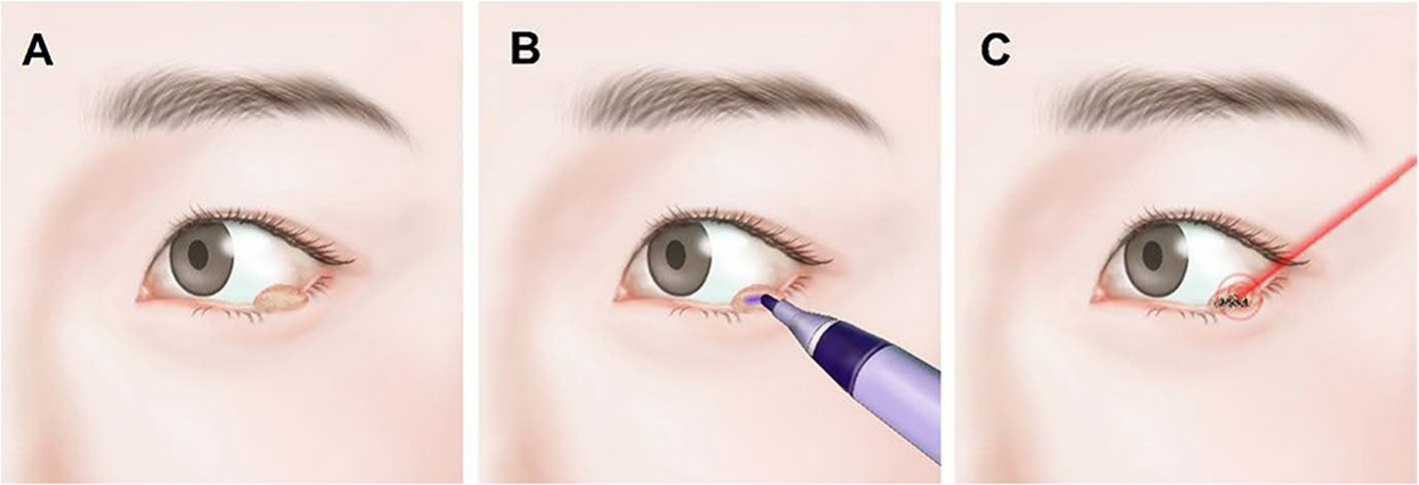
Argon laser photoablation using a violet marker for benign tumors of the eyelid. **A** Left lower eyelid mass **B** A violet marker was applied to the eyelid mass. **C** The painted lesion is then treated with the argon laser

The procedure is presented in Fig. 1 and S1 Video. The eyelid lesion was first anesthetized with 2 ml of 2% lidocaine and 1:100,000 epinephrine. A violet skin marker pen (Skin Marker, Viscot Medical, LLC, NJ, USA) was applied to the entire surface of the eyelid mass (Fig. 1B), thereby, the uptake of argon laser energy into the eyelid mass was increased. Hence, the required laser power was reduced and damages to surrounding tissue were reduced. The laser beam was focused directly on the tumor, and laser spots were then applied (Fig. 1C). In cases in which the eyelid margin was involved, corneal protection (Cornea shield, Ellman, NY, USA) was set in front of the cornea. Eyelids were stretched and everted whenever necessary. The treated area covered the entire mass. An average of 312 spots (range 45 to 1,133) were applied with an argon green laser (VISULAS 532 s, Carl Zeiss Meditec, Jena, Germany) using a spot size ranging from 150 to 500 μm, a power of 200 to 400 mW, and a spot duration between 0.1 and 0.2 s. Whenever the size of the lesion was large enough, a pathologic confirmation was done. The lesion was grasped with forceps when the argon laser was applied to the base of the lesion. The excised tissue was retained for histopathological examination if there was enough tissue. After the treatment, ophthalmic ointment including Neomycin, Polymyxin B, and Dexamethasone Ophthalmic (Maxitrol, Alcon, Fort Worth, TX) were applied topically at bedtime for 7 days. All procedures were performed by one skilled surgeon (H.J.S.). Patients had regular follow-up examinations at 1 week, 1 month, 3 months, and 6 months after the procedure. After 6 months the patients were asked to visit when they felt that they needed to.

## Results

Clinical data of included eyes are listed in Table 1. 19 patients (10 men and 9 women, mean age 58.7 years) were followed up for an average of 10.5 months after the procedure. The mean lesion size was 4.82 mm. Treatment was well tolerated by all patients. The lesions were primarily located on the lower eyelid (58%). Postoperatively, 11 lesions could be examined histologically. Most of the tumors were nevi or papilloma (Table 2). Three nevi, two verrucae, two seborrheic keratoses, and one retention cyst could be diagnosed only by clinical appearance, which was then supported by the findings during laser treatment.

**Table 1:** Demographic and preoperative characteristics

Characteristics	*n* = 19 (19 eyelids)
Age, years (mean ± SD)	48.6 (range, 24–70)
Sex (male / female)	10 / 9
Laterality (right / left)	10 / 9
Location (upper / lower / canthus)	6 / 11 / 2
Size of lesion (≤ 5 mm / > 5 mm)	12 / 7
Postoperative complication^a^ and recurrence, %	0 (0%)
Mean follow up (month)	10.5 (range, 6–18)

^a^Eyelid malposition, contour deformity, ocular irritation, and cosmetic dissatisfaction

SD, standard deviation

**Table 2:** Histology of 11 Argon Laser-Treated eyelid tumor of 19 treated lesion^a^

Lesion type	No
Dermal nevus	4
Papilloma	3
Seborrheic keratosis	2
Molluscum contagiosum	1
Cyst	1

^a^Seven tumors could be diagnosed only by clinical aspects

**Fig. 2:**
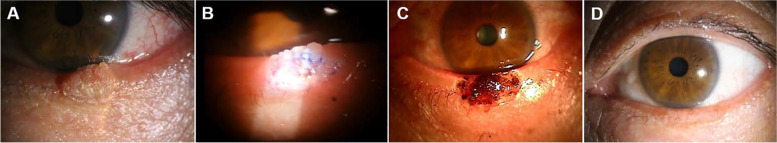
A 64-year-old man had a large eyelid mass (8 mm) on the left side. The eyelid mass was successfully removed with one session of argon laser photoablation. **A** Preoperative appearance. **B** Painted mass. **C** Appearance immediately after argon laser photoablation (S1 Video). **D** One-month postoperative appearance

Our novel approach required very little time (< 5 min) and caused very little discomfort to the patients after laser treatment. During laser treatment, no complications were observed. Only one patient complained of pain during treatment. The wounds were dry after argon laser therapy and the wounds were completely epithelialized after 2 to 4 weeks. The treated areas further improved cosmetically over the following 1 to 2 months and were finally covered by a normal-appearing epithelium (Fig. 2). During the follow-up, remarkable notches, neovascularization, and hyper/hypopigmentation never occurred. There were no occurrences of ocular or palpebral complications such as infections, secondary hemorrhages, failure to heal, corneal irritation, entropion, or ectropion. In all cases, the final cosmetic and anatomical results were good after just a single session of laser treatment. All patients were satisfied with the cosmetic results. No recurrence occurred during the follow-up period.

## Discussion

Since argon lasers have been successfully used for dermatological diseases [12], ocular use is being increasingly considered by ophthalmologists in various disorders of the anterior eye segment and ocular adnexae, such as xanthelasma palpebrarum [13] and trichiasis [14]. In our patient cohort, the mean mass size was 4.82 mm, and mean follow-up period was 10.5 months. Argon laser treatment with violet marker under slit-lamp resulted in complete resolution of the eyelid mass in all patients without recurrence or complications in a single session treatment. Treatment was well tolerated by all patients regardless of tumor size.

Our laser technique is different from the traditional technique by using lower laser power and increasing the absorption rate of laser energy with a marker. Wohrlab et al. [10] used laser power from 410 to 6100 mW; Schellini et al. [5] used the laser with 500 μm spot sizes and a laser power between 1000 and 1200 mW. Higher laser energies (> 1000 mW) with larger spot sizes (> 500 μm), however, may cause retractions in the normal adjacent skin and may result in scar formation [4]. In our study, we used the argon laser with a spot size of 150–500 μm and a power between 200 and 400 mW.

**Fig. 3:**
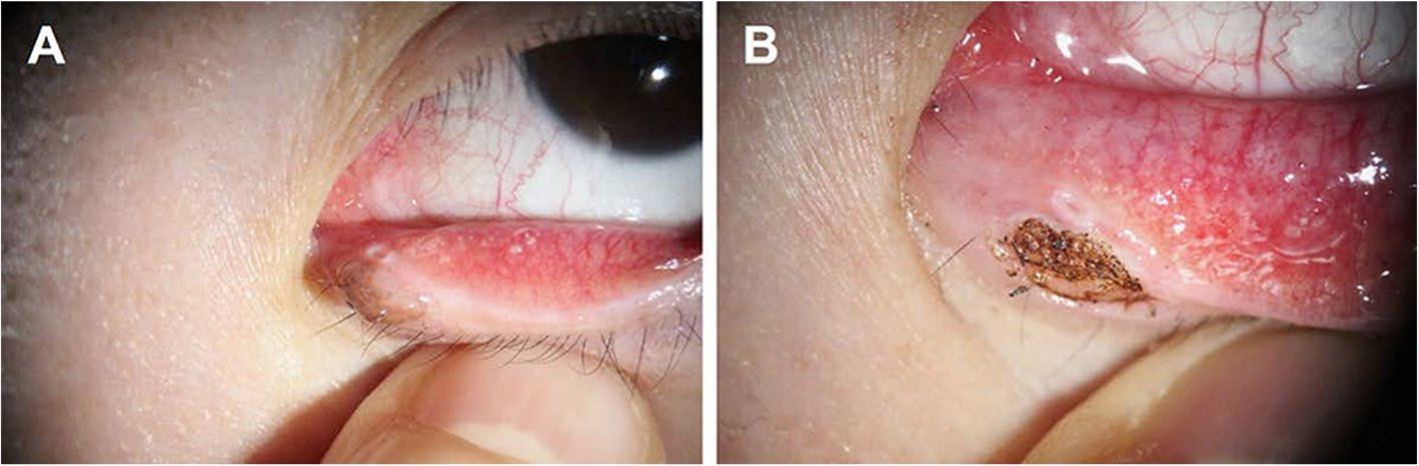
A 56-year-old women had a left eyelid mass (4 mm) close to the lacrimal punctum, which was successfully removed in a single session of modified argon laser treatment. **A** Preoperative appearance. **B** Immediate postoperative appearance

**Fig. 4:**
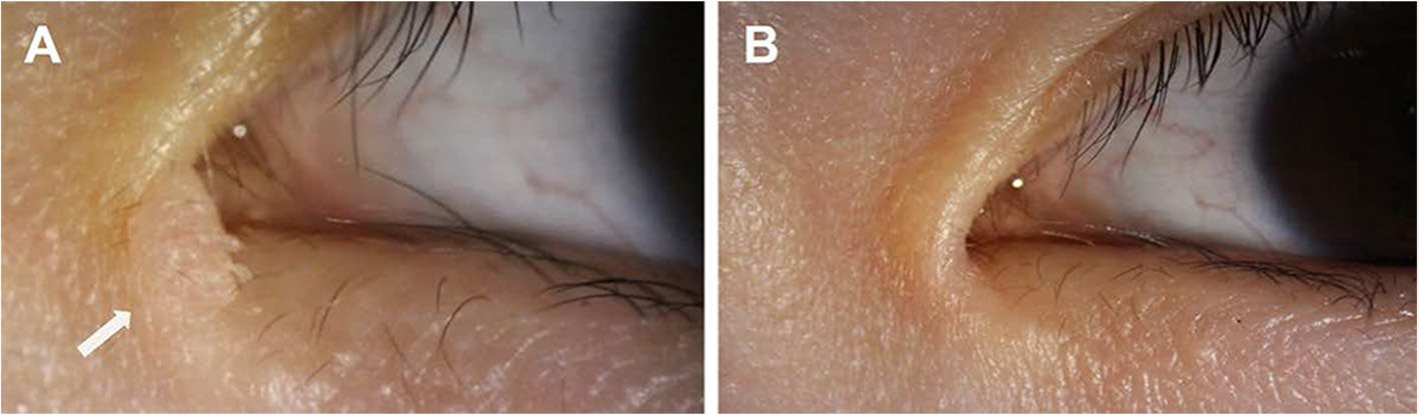
A 34-year-old women had a left eyelid mass (4 mm) in the medial canthus that was successfully removed in a single session of argon laser photoablation. **A** Preoperative appearance. **B** One-month postoperative appearance

Our modified argon-laser method—which involves staining the surface of eyelid mass with a dark-purple marker in order to efficiently transfer the laser energy to the underlying tissue—is also effective for treating lesions with a larger size (> 5 mm) with a relatively low dose of energy. Furthermore, the use of slit-lamp biomicroscopy during laser treatment increases the accuracy of treatment, and hence it facilitates the selective ablation of targeted tissue while reducing unwanted damage to healthy tissue [4, 5]. This is especially advantageous in eyelid masses that are located in locations difficult to treat surgically without compromising other structures, like the canaliculus (Figs. 3 and 4).

The emission peaks of the argon laser are 488 nm (blue) and 514 nm (green); this wavelength distribution overlaps with the absorption spectrum of melanin pigment [15]. When such a laser is irradiated to a lesion without melanin pigment, a higher laser power is required to deliver the same amount of energy, which increases pain and treatment time, and has the potential to damage adjacent tissues. To address these issues, we painted the eyelid mass surface with a dark purple marker so that laser energy could be effectively delivered to the mass and cyst. This method was used in a previous study in our group to increase the absorption of argon laser energy when performing conjunctival cyst removal with argon laser [11]. Even if the laser accidentally reaches the adjacent tissue, it is especially absorbed in the marked area. Thus, the laser energy is mainly applied just to the desired area. Our results are consistent with previous studies which demonstrated that treatment with argon lasers is an effective and safe method in the treatment of benign lesions of the eyelid. Schellini et al. [5] reported no postoperative keloid or hypertrophic scar formation, so does our study. The advantages of the laser procedure, compared to surgery in benign lesions are numerous: magnified view of the tissue removal allowing a complete excision and avoiding unwanted damage to adjacent structures like hair follicles and punctum; good hemostasis and consequently preventing postoperative swelling and bruise, avoiding wound sutures and bandage; fast and painless technique; outpatient office procedure with little post-operative care; good acceptance of the technique by most of the patients; more cost-effective than removing the eyelid tumor surgically in the operating room [10, 16].

Argon laser therapy of benign eyelid tumors results in very satisfactory wound healing, which is consistent with the previous finding by Wohlrab et al. [10]. In our cohort, eyelid margin showed a well-healed appearance after laser tumor ablation without any surgical intervention. We postulated that the tumor destroyed by heat energy from the argon laser became a necrotic material, and then the eyelid heals by laissez faire. Laissez faire often leads to a good healing process in the periorbital area, especially in the lower eyelid. Laissez-faire also has more advantages than reconstruction for tumor surveillance [17].

Another advantage of our method is that it is easily repeatable and can be applied to recurrent benign lesions such as recurrent eyelid papilloma. Also, despite the laser photoablation, pathologic examination was possible by taking out some tissue with forceps during laser treatment (S1 Video). A previous study by Wohlrab et al. [10] documented that eyelid mass removal with the argon laser does not alter the tissue in such a way that it prevents accurate histopathologic examination. Hence, an incisional biopsy before treatment does not seem to be necessary. In addition, argon laser benign eyelid excision does not require facilities related to surgery and is a time-saving, option. The procedure can also be performed on patients who are afraid of surgery or cannot lie down on the operating table (e.g. due to spinal deformities). Since there are no sutures required, frequent follow-up visits are not necessary [4].

However, it should be noted that argon laser treatment should be limited to benign lesions. Careful attention should be paid to patient selection, especially when a potential malignancy or malignant transformation cannot be fully excluded. For an accurate diagnosis, a careful assessment of the history would be mandatory. Suspicious lesions should be treated with conventional excisional biopsy. Although we performed a biopsy of the eyelid lesion during the laser procedure, histologic confirmation in flat lesions or small retention cysts and hemangioma may sometimes not be possible because there is not always enough tissue left [10]. In addition, even if the lesion looks benign, there is a possibility of malignancy. Thus a certain period of follow-up would be necessary to check for a recurrence or malignant transformation after the laser procedure. In this regard, considering a biopsy first and performing a laser treatment later when a benign lesion is confirmed would be safer praxis.

Our method cannot be used on patients who cannot maintain a sitting position, nor on patients who cannot cooperate. Another limitation of our modified laser treatment is that histopathological examination was not possible in every case. Our study has a retrospective nature and a small number of patients. Prospective studies with a larger number of patients and long-term follow-up are needed to validate our experimental procedure according to tumor size. Also, further studies comparing surgical techniques might be necessary.

In conclusion, our modified method of benign eyelid tumor removal with an argon laser utilizing a violet marker is easy to perform and reduces the damage to surrounding tissue. Our method can be applied to relatively large lesions, and the success rate is not inferior to that of the surgical method. Our method is also easier to learn and less expensive than conventional surgery. Also, slit-lamp biomicroscopy with an argon laser enables more-delicate control and the selective ablation of the targeted tissue and reduces unwanted damage to healthy tissue. Especially this technique has more advantages than surgery in tumors near the punctum. Further studies including more patients and comparing various types of benign tumors might be necessary.

## Supplementary Information

**Additional file 1:** **S1 Video. **A case of modified argon laser photoablation for benign tumors of the eyelid.

## Data Availability

The datasets used and/or analyzed during the current study are available from the corresponding author on reasonable request.

## Declarations

### Availability of data and materials

The datasets generated and/or analysed during the current study are not publicly available due to human data but are available from the corresponding author on reasonable request.
